# Faith, Science, and Choice: Vaccine Attitudes Among Religious University Students

**DOI:** 10.3390/vaccines14060546

**Published:** 2026-06-20

**Authors:** Isaiah Aduse-Poku, Keersty J. B. Thompson, Afton Fillmore, Leah Sim, Isaac A. Woolley, Elizabeth G. Bailey, Brian D. Poole, Jamie L. Jensen

**Affiliations:** 1 Department of Biology, Brigham Young University, 4102 Life Sciences Building, Provo, UT 84602, USA; aduse@student.byu.edu (I.A.-P.); kjb259@byu.edu (K.J.B.T.); siml04@byu.edu (L.S.); iwoolley@byu.edu (I.A.W.); liz_bailey@byu.edu (E.G.B.); 2 Plant and Wildlife Sciences Department, Brigham Young University, 4102 Life Sciences Building, Provo, UT 84602, USA; amf04@byu.edu; 3 Department of Microbiology and Molecular Biology, Brigham Young University, Provo, UT 84602, USA; brian_poole@byu.edu; 4 Department of Preparatory Learning, School of Medicine, Brigham Young University, Provo, UT 84602, USA

**Keywords:** vaccination attitudes, vaccine hesitancy, Q-methodology, college students, faith-based university, identity-protective cognition

## Abstract

Background/Objectives: Vaccine attitudes are an individual’s beliefs, feelings, and evaluations regarding vaccines. Limited research has examined how students in faith-based university settings organize these attitudes. This study looked at vaccination attitudes among students at a religious university where faith, science, family, and politics often influence how students think and make decisions. Methods: This study used Q-methodology to examine shared viewpoints about vaccination. A concourse of 240 statements was developed from published literature, public discourse, and student interviews, then reduced to a 37-statement-Q-set. Undergraduate students enrolled in an introductory nonmajors biology course completed digital Q-sorts. We analyzed the data using by-person factor analysis, along with principal components analysis and Varimax rotation. Follow-up interviews helped us interpret the factors. Results: Three viewpoints explained 59% of the study variance. The first viewpoint, Faith-Integrated Institutional Trust, showed strong trust in science, public health agencies, and religious leaders. People in this group saw vaccination as both a moral duty and a way to protect others. The second viewpoint, Skeptical Autonomy and Institutional Distrust, emphasized personal choice, family influence, and distrust of government and official vaccine information. The third viewpoint, Pragmatic Autonomy and Science Confidence, endorsed vaccines and scientific evidence while also prioritizing individual decision-making over mandates. Conclusions: Science alone does not explain vaccination attitudes among college students. Trust, identity, and personal autonomy also play an important role. Vaccine communication should therefore connect scientific evidence with students’ moral commitments, trusted relationships, and concerns about freedom, especially in settings where faith influences health decision-making.

## 1. Introduction

Vaccination remains one of the most effective public health interventions, with strong evidence for large reductions in mortality from infectious diseases [1,2,3]. Despite these gains, vaccine uptake has stagnated or declined in many settings, and outbreaks of measles, pertussis, and other vaccine-preventable diseases have re-emerged [4]. In 2024, the World Health Organization (WHO) warned that increasing measles incidence in Europe indicated a rising global risk and that more than half of countries worldwide could face a major outbreak that year [5]. In the United Kingdom, childhood coverage for measles and pertussis has dropped below the WHO 95% target, alongside renewed outbreaks and growing case numbers in vulnerable groups [6,7].

Kiang et al. [8] modeled large health losses under scenarios of reduced childhood vaccination coverage in the United States. Their simulations suggest that current state-level immunization rates may already be low enough for measles to potentially regain endemic status, a trend that could be reversed only by increasing vaccine uptake. In a scenario where childhood vaccination levels fall by 50%, the model projects severe nationwide impacts over 25 years, including about 51 million measles cases, nearly 10 million rubella cases, more than 4 million poliomyelitis cases, almost 200 diphtheria cases, around 10 million associated hospitalizations, and over 159,000 deaths. These projections indicate that maintaining high vaccination coverage is necessary to prevent the return of previously eliminated diseases and associated morbidity and mortality [8].

Maintaining high vaccination coverage requires attention to the full range of vaccine attitudes. Vaccine attitudes describe individuals’ beliefs, feelings, and evaluations regarding vaccination, including how they accept, question, delay, or refuse vaccines [9,10,11,12,13,14,15,16] Because vaccine attitudes, including hesitancy, are context-specific and vary by setting, time, and vaccine type [15], research must examine the social, cultural, religious, and institutional factors that shape how individuals organize their views about vaccination.

Surveys in the United States and other countries show that young adults and college students have been among the groups most reluctant to receive COVID-19 vaccines [17,18,19,20]. In a multi-university survey conducted in spring 2021, 57.3% of college students reported having received a COVID-19 vaccine, while 29.0% were classified as vaccine-hesitant [18]. Relatively low uptake also appears for other recommended vaccines. In a study of HPV vaccination among young adults in a southern U.S. state, 62.4% of 1708 respondents had initiated the HPV vaccine series, but only 28.8% completed all recommended doses. Among those who initiated the series, 45.1% reported full completion [21]. Similarly, in a study of more than 1600 respondents at a Midwestern university, only about half reported receiving a COVID-19 vaccine. Among unvaccinated respondents, 49% reported no intention to get vaccinated and 22% were undecided [22].

College students’ attitudes about vaccinations vary depending on race, gender, locality, education, and culture [23,24,25,26,27]. Additionally, rampant misinformation about vaccination, especially during the COVID-19 pandemic, is highly accessible due to widespread use of social media, contributing to general hesitancy [25]. Students living in more conservative and rural areas tend to be more vaccine-hesitant than those living in urban areas [26,27]. Level of education also plays a role in the decision to vaccinate [23,26]. Students with higher levels of education, such as college upperclassmen and master’s students, are more likely to be knowledgeable about vaccines and more willing to receive them [23,26]. College-aged people are less likely to get vaccinated if they receive medical advice from family or have a lower level of education [23,27].

Group identities also shape vaccination attitudes, especially political and religious identities. Among college students, these identity-linked processes appear in patterns of hesitancy and uptake. Prior research has found higher hesitancy among students with moderate or conservative political orientations and lower hesitancy among students with stronger collectivist values, while individualism showed no association [28]. Best et al. [29] found that sexual activity fully mediated the relationship between religious/spiritual beliefs and HPV vaccination among college women. Examining individuals’ attitudes toward vaccination is important for understanding how they evaluate risks and benefits, negotiate trust in scientific and institutional authority, and ultimately decide whether to accept or decline recommended immunization [30,31,32,33,34,35]. Exploring what influences young adults’ vaccination decisions is important, as this population is still forming lifelong health behaviors. In addition, they are beginning to assume independent responsibility for medical choices that can influence disease transmission in campus environments as well as in future family and civic contexts.

Given the recent epidemiological trends among young adults and college students and the context-specific nature of vaccine attitudes, it is important to examine how religious college students describe and organize their views and opinions about vaccines and vaccination within a particular institutional and cultural environment [36,37]. This study examines students at Brigham Young University (BYU), a large private religious institution in the western United States. BYU was selected because it provides an information-rich setting for examining vaccine attitudes at the intersection of faith, science education, family influence, political identity, and institutional trust. The university combines scientific literacy expectations in its general education curriculum with strong communal and faith-based identity commitments. The sponsoring religious organization, the Church of Jesus Christ of Latter-day Saints, has expressed clear support for vaccination, including the COVID vaccine; however, members are encouraged to be “responsible to make their own decisions about vaccination” [37], and many members express vaccine hesitancy associated with conservative ideologies (e.g., [38]). Many students at BYU report conservative political views, strong religious affiliation, and close ties to family and congregation networks [39]. This sociocultural context makes BYU a useful setting for examining how religious, cultural, and political identities interact with scientific reasoning in shaping vaccine attitudes. It also allows analysis of how messages from church leaders, family networks, public health authorities, university faculty, popular media, and social media converge or compete in students’ vaccine decision-making.

In this study, we used Q-methodology to explore the perspectives of undergraduate students at BYU. Q-methodology is a framework that identifies shared viewpoints by analyzing complete sorting patterns across participants. Prior research has used Q-methodology to examine subjective meaning-making around vaccination and other health topics, including vaccine hesitancy, in diverse populations [40,41]. To the best of our knowledge, no prior study has applied Q-methodology to vaccine attitudes among college students in a faith-based setting.

This study addresses that gap by examining how undergraduates in a faith-based educational institution organize their attitudes toward vaccination. We focused on the research question: What patterns of vaccination attitudes emerge among these students? We then used these patterns to identify distinct subjectivities and value orientations that influence vaccine decision-making within this faith-based context. We conclude with a discussion of ways to improve vaccine communication and education so that they resonate with students’ identities and support vaccine acceptance.

### 1.1. Rationale for Using Q-Methodology

Q-methodology combines qualitative and quantitative approaches to study subjectivity and preference by identifying shared viewpoints within a group [42]. It examines how individuals understand specific topics through Q-sorts, in which they rank a set of statements on a fixed, quasi-normal grid according to the degree to which they agree or disagree with those statements [43]. A defining feature of Q-methodology is that it reverses the logic of conventional factor analysis. Instead of treating survey items as variables and participants as the sample, Q-methodology treats the respondent-sorted statements as the sample and the participants as the variables. It then calculates correlations between participants based on how similarly they sort the statements, rather than correlating items across participants. This process groups respondents into factors, with each factor representing a distinct and internally coherent viewpoint or pattern of subjectivity [43,44].

We used Q-methodology for three main reasons. First, vaccine attitudes are highly contested and often polarized, especially since the COVID-19 pandemic [45,46]. Q-methodology is well suited to topics that evoke strong views and ethically sensitive reactions because it asks participants to sort pre-established statements rather than respond to direct, potentially confrontational questions [47,48,49]. In the BYU context, where social, religious, and political identities intersect, we needed an approach that could capture a wide range of subjective positions without placing students into researcher-defined categories [42].

Second, prior Q studies in health and vaccination suggest that individuals’ positions regarding vaccination often cluster into coherent, internally consistent viewpoints rather than appearing as isolated opinions [40,41]. In Q-methodology, factors represent groups of participants who rank the same set of statements in similar ways and therefore reveal a shared perspective [43,49]. Such shared perspectives become visible through the forced sorting task, which structures participants’ responses while still allowing them to prioritize among competing considerations according to their own viewpoints. Because participants must weigh each statement in relation to all others, the process encourages more careful and reflective engagement with their responses [50]. In this way, Q-methodology allowed us to systematically examine students’ belief patterns and compare the distinct viewpoints that emerged across the participant group.

Third, Q-methodology offers a rigorous and transparent design that fits well with public health and education research. Because all participants sort the same predefined set of statements, the method supports careful comparison across individual frames of meaning while preserving each participant’s viewpoint [48,49]. In Q-methodology, the participant set (P-set) is constructed to maximize the diversity of viewpoints rather than to achieve statistical representativeness [49]. Given our interest in how BYU students integrate faith, science, and identity in their opinions about vaccination, this focus on depth and patterned diversity was more appropriate than a large-scale survey. Q-methodology allows us to describe the shared viewpoints that organize students’ vaccination attitudes, while acknowledging that these factors are context-specific and not statistically generalizable beyond the study setting.

### 1.2. Ethical Approval

This study received approval from the Institutional Review Board at the primary author’s institution, Protocol IRB2025-145. All participants provided electronic informed consent before completing the survey and interviews.

## 2. Methods

Prior studies have used Q-methodological procedures in different contexts and subject areas [51,52,53]. In line with these procedures, we followed a six-stage Q-method protocol. First, we developed a concourse, which is the broad collection of viewpoints, opinions, and expressions about vaccination drawn from sources such as literature, public discourse, and interviews. Second, we constructed the Q-set, which is the smaller set of statements selected from the concourse for participants to sort. Third, we selected the P-set, or participant group. Fourth, participants sorted the Q-set statements into a quasi-normal distribution grid according to their viewpoints. Fifth, we analyzed the completed Q-sorts using casewise factor analysis. Sixth, we interpreted the resulting factors and their defining statements.

### 2.1. Development of the Concourse

Following Watts and Stenner [49], we defined the concourse as the broad field of shared viewpoints and meanings about vaccination from which we could draw a context-specific set of statements. To capture the range of viewpoints needed for our studies, we developed the concourse from multiple sources. These included a comprehensive review of vaccines and vaccination research, and statements from the media and other public sources that discussed vaccines in health, religious, political, and educational contexts. These sources were supplemented by interviews with six randomly recruited BYU undergraduates. The purpose of these interviews was to confirm whether the statements already gathered were culturally relevant and familiar to our target population of undergraduates in a faith-based university setting. Eleven undergraduate research assistants, including four student co-authors, helped refine the initial pool of statements by removing redundant, unclear, or incomplete items. These assistants were separate from the six undergraduate interview participants noted above. The final concourse included 240 statements covering a wide range of views about vaccination. This set of statements served as the sampling frame for constructing the Q-set.

### 2.2. Development of the Q-Set

The lead author (I.A.-P.) and one co-author (K.J.B.T.) conducted thematic coding of the 240 concourse statements and selected 60 provisional statements following Q-methodological guidance on coverage and balance [49]. To make the reduction process systematic, we used a coding framework that organized statements by content domain and attitudinal position. Content domains included general evaluations of vaccination, trust in science and government agencies, family influence, religious and cultural values, media and social media, misinformation and myths, perceived risks and benefits, mandates, personal freedom, and community responsibility. Within each domain, statements were also reviewed for their position on the vaccine-attitudes spectrum, including acceptance, confidence, urgency, ambivalence, hesitancy, skepticism, and refusal.

We selected statements that provided broad coverage across these domains while also representing contrasting positions within the vaccine-attitudes spectrum. This process helped minimize overlap and reduced the chance that the Q-set would overrepresent vaccine hesitancy, skepticism, or any single stance. We also balanced positive and negative wording to support careful sorting and reduce socially desirable responding. Statements were excluded when they repeated an idea already represented more clearly by another item, addressed a point outside the study focus, used leading language, or contained technical jargon, double-barreled phrasing, or ambiguous wording. The coding framework and examples of included and excluded statements are provided in Supplementary Tables S3–S5.

Subject experts in biology education and vaccination reviewed the 60-item Q-set for content relevance and clarity. Following expert review, the lead author (I.A.-P.), co-author (K.J.B.T.), and subject experts reached consensus on a final set of 37 statements, which formed the Q-set (see Supplementary Information). We kept the Q-set at 37 items so participants could complete the sorting task within class time and without undue burden, while still preserving coverage of the major content domains. This Q-set was then pilot tested with a small group of seven students similar to the target population. Pilot feedback informed minor wording changes and the addition or adjustment of items where coverage seemed limited. This was done without changing the intended meaning of the statements. Although the final Q-set of 37 statements was slightly below the often-cited range of 40 to 80 items, it falls within accepted practice for Q studies focused on a defined topic and a relatively homogeneous participant group [49].

### 2.3. Selection of P-Set (Participants)

In Q-methodology, careful construction of the participant group, or P-set, is important to the quality of the viewpoints that emerge. Because Q-methodology aims to identify a range of perspectives within a specific group rather than estimate population prevalence [54], we used purposive recruitment to access a participant pool likely to contain varied viewpoints on vaccination. We recruited participants from an introductory nonmajors biology course at BYU during the Fall 2025 semester. This course provided an appropriate recruitment setting because it enrolls a large and diverse mix of students across majors and backgrounds and as a general education requirement for many students across the university.

### 2.4. Q-Sorting (Participant Task)

Before the Q-sort, we sent invitations to all students enrolled in the course and invited them to participate. At the beginning of the activity, students received an overview of the project aims, a brief introduction to Q methodology, and an explanation of how the findings would be reported. All participants provided written informed consent, and we anonymized the data so participant identity remained private. Aside from gender, academic major, political affiliation, and religious affiliation, we did not collect sensitive personal health information or other special-category data.

Because we wanted our participants to give careful attention to the sorting task, we implemented the Q-sort as a voluntary class activity and invited all enrolled students (*n* = 210) to complete the task. Participants completed the Q-sort digitally using Qsortware, an online platform developed by Pruneddu and Zentner [55]. This Q-sort platform has been used in previous Q studies [55,56] to achieve quality results. Qsortware provides a web-based interface that mimics a physical Q-sort and allows participants to drag and drop statements into a forced-choice distribution [55].

**Figure 1:**
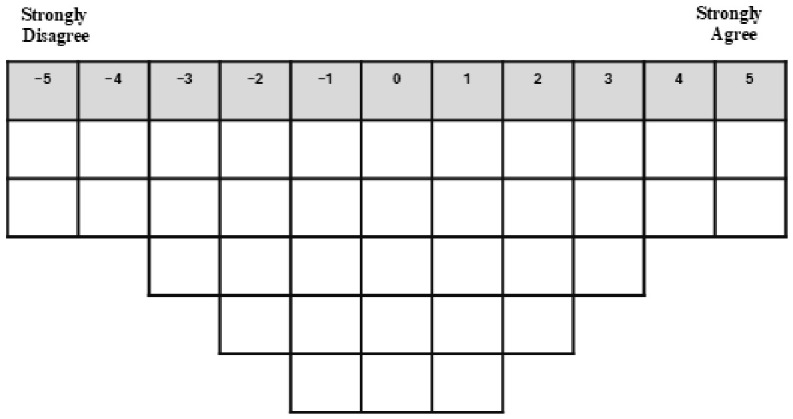
Q-sort grid.

Participants first read through all 37 statements and completed a brief pre-sort into three broad categories: agree, disagree, and neutral. After this initial sort, participants were introduced to the forced quasi-normal grid and asked to rank the statements on a scale from −5 to +5, with −5 meaning “most disagree” and +5 meaning “most agree,” and 0 indicating uncertainty or neutrality. Each box on the grid could hold only one statement, so participants had to rank the statements relative to one another rather than rate each one independently (Figure 1). This forced distribution, which required participants to position each statement relative to all others, is consistent with Q-methodology’s emphasis on comparative judgment [57]. There was no correct sorting pattern, because each configuration represented the participant’s own point of view. Q-sortware allowed participants to adjust their placements until they felt the final arrangement best reflected their perspective.

Because participation in the Q-sort was voluntary, all students enrolled in the course (*n*= 210) were invited to complete the task, and 160 students completed and submitted responses. Missing responses resulted from students being absent that day or not fully engaging in class activities. We did not gather any attitudinal data from students who did not complete the sort so we do not know if they represented a significant alternative unmeasured factor, but we have no reason to suspect that they do. From these 160 completed Q-sorts, we constructed a smaller set of 30 sorts for formal analysis. Since increasing sample size beyond what is needed to identify the main viewpoints present does not add analytic advantage in Q-methodological practice, we prioritized diversity and data quality rather than maximizing numbers [39,53]. We used a multi-step screening process to select a diverse set of high-quality participant Q-sorts for statistical analysis. The final Q-sorts were selected randomly within the stratified framework rather than by convenience. We also maintained a clear record of all changes from the initial Q-sort pool to the final selected Q-sorts, as described below. A total of 160 participants completed Q-sorts. Of these, 96 identified as male and 61 as female, with a small number of missing or unclassified responses. Among male participants, 51 identified as conservative, 5 as liberal, 30 reported no political affiliation, and 10 identified as independent. Among female participants, 24 identified as conservative, 6 as liberal, 28 reported no political affiliation, and 3 identified as independent. We screened the initial 160 sorts for analytic eligibility. This was done by removing responses that were missing key demographic information needed for stratified sampling or had placeholder entries. We treated blank answers and entries such as N/A as missing. Using this rule, 66 of the 160 responses were missing at least one key demographic field used for stratified sampling. The counts for missing academic major, gender, political leaning, and religious affiliation overlap because some responses were missing more than one field. Missing or placeholder entries appeared in academic major (25), gender (4), political leaning (54), and religion (5). After removing cases with incomplete stratification data, 94 Q-sorts remained eligible for sampling. We then reviewed participants’ written comments about their completed sorts as an additional quality check. Sorts were removed when participants explicitly stated that their final sort did not accurately represent their vaccination attitudes, admitted that they placed statements randomly, or indicated that they did not understand the sorting task. After applying this additional quality check, 77 Q-sorts remained eligible for sampling. We used the 77 eligible Q-sorts, rather than a researcher-defined target distribution, as the sampling frame because our goal was to preserve the demographic diversity present in the analytically usable pool. We selected a final 30 Q-sorts using proportionate stratified random sampling across academic major, gender, religious affiliation, and political leaning. As a sensitivity check, we analyzed the remaining completed Q-sorts that were not included in the final 30-sort analytic sample. This comparison produced three factors with a highly similar factor structure, suggesting that the final analytic sample preserved the main viewpoints present in the larger completed Q-sort pool.

### 2.5. Interviews

Following preliminary analysis of the Q-sorts, we invited a subset of 10 participants to complete follow-up interviews (see Supplementary Table S2). These interviews were used as post-sort interpretive support rather than as a separate qualitative analysis. Interview participants were selected from among those whose Q-sorts loaded significantly and strongly on the retained factors. We also considered demographic distribution when making these selections so that the interview sample included participants from different demographic backgrounds across the factor structure. The interviews examined the reasoning behind participants’ rankings, with particular attention to statements placed at the extremes of the distribution. We asked participants to explain why they strongly agreed or disagreed with specific statements, how their beliefs and experiences shaped their placements, and whether any statements were difficult to rank.

### 2.6. Analysis

We intercorrelated the 30 completed Q-sorts and conducted a by-person factor analysis using KADE software, version 1.3.1. The Q-sort data were organized using the Ken-Q version 2 Excel template before analysis [58]. The analysis followed standard Q-methodological procedures and proceeded through three main transitions: from Q-sorts to factors, from factors to factor arrays, and from factor arrays to interpretation.

#### 2.6.1. From Q-Sorts to Factors

We began the analysis by calculating a correlation matrix. In Q-methodology, correlation provides a “measure of the nature and extent of the relationship between any two Q-sorts and hence a measure of their similarity or otherwise” [49]. Each of the 30 Q-sorts in our study represented a participant’s configuration of 37 vaccine-attitude statements placed on a forced distribution grid ranging from −5 to +5. We correlated each Q-sort with every other Q-sort to produce a 30 × 30 correlation matrix.

We then conducted factor analysis on the intercorrelations using principal components analysis (PCA) as the extraction method. We applied Varimax rotation, rather than judgmental rotation, to obtain orthogonal and interpretable factors, because we did not have a predefined hypothesis about the factor structure [59]. To ensure that only Q-sorts that clearly reflected a factor’s shared configuration contributed to its definition, we treated factor loadings as significant at *p* < 0.05. We treated each significantly loading Q-sort as a factor exemplar (these participants ranked the statements in highly similar ways and therefore shared a comparable viewpoint).

#### 2.6.2. Factor Retention

**Figure 2:**
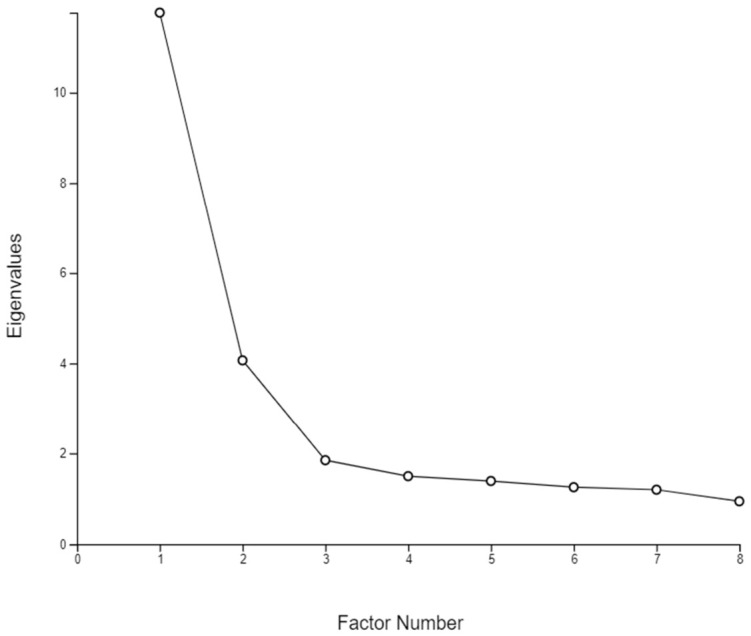
Scree Plot of eigenvalues across factors.

Principal components analysis initially extracted eight factors. We determined factor retention using three complementary criteria: the Kaiser criterion (eigenvalues greater than 1.00), inspection of the scree plot for an elbow point, and theoretical interpretability [49,60]. Seven factors exceeded the eigenvalue threshold (Factor 1 = 11.77, Factor 2 = 4.07, Factor 3 = 1.85, Factor 4 = 1.50, Factor 5 = 1.39, Factor 6 = 1.25, Factor 7 = 1.20) (Table 1). Although seven factors exceeded the eigenvalue threshold, we retained three factors for interpretation because the scree plot (Figure 2) showed a clear inflection after the third factor, followed by a flatter curve. This indicated that Factors 4 through 8 offered limited additional explanatory value and were therefore not named or retained for interpretation.

The first three factors together accounted for 59% of the total variance (39%, 14%, and 6%, respectively) (Table 1). This level of explained variance is appropriate for Q-methodological research, which prioritizes interpretable viewpoint structures over maximizing explained variance [49].

#### 2.6.3. From Factors to Factor Analysis

**Figure 3:**
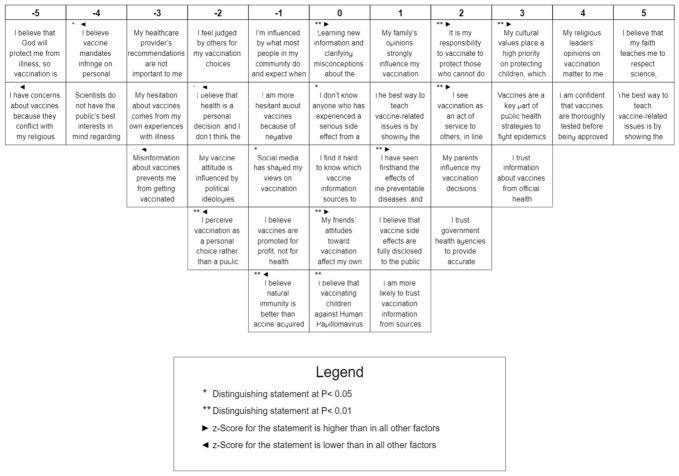
Composite Qsort array for Factor 1, “Faith-Integrated Institutional Trust.” Columns represent the fixed Q distribution from −5 (strong disagreement) to +5 (strong agreement).

**Figure 4:**
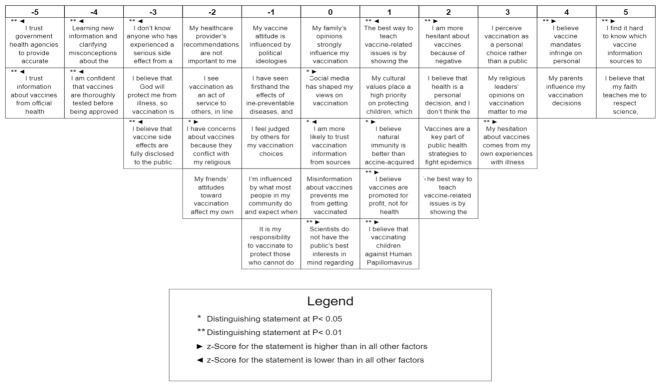
Composite Qsort array for Factor 2, “Skeptical Autonomy and Institutional Distrust.” Columns represent the fixed Q distribution from −5 (strong disagreement) to +5 (strong agreement).

**Figure 5:**
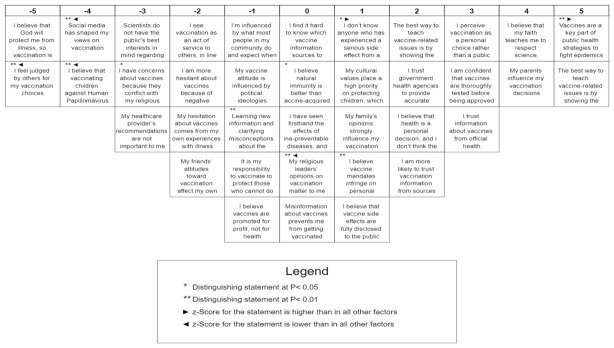
Composite Qsort array for Factor 3, “Pragmatic Autonomy and Science Confidence.” Columns represent the fixed Q distribution from −5 (strong disagreement) to +5 (strong agreement).

Next, we converted the extracted factors into factor arrays, which are ideal-typical Q-sorts that represent each viewpoint. For each factor, we computed a weighted average of the defining Q-sorts, giving greater weight to participants with stronger factor loadings. The resulting factor arrays are composite Q-sorts that capture the characteristic ranking pattern for each shared perspective. Each array retained the full Q-set structure, with statement scores ranging from −5 (strong disagreement) to +5 (strong agreement) (Figure 3, Figure 4 and Figure 5).

#### 2.6.4. From Factor Analysis to Factor Interpretations

The factor arrays served as the primary basis for interpretation. We read each array as a whole and attended to the complete pattern of scores from −5 to +5 rather than focusing only on the most extreme statements [49,57]. This whole-array approach treats the configuration of scores as a single, structured viewpoint.

We began the factor interpretation by identifying the statements placed at ±5 in each array, because these items marked what participants associated with each factor most strongly endorsed or most strongly rejected. The reading then moved stepwise inward to items at ±4, ±3, then to items at ±2, ±1, and 0. To avoid treating scores around 0 as simple neutrality, special attention went to mid-ranked statements whose placement suggested qualified support, tension, or consensus, especially when the same items appeared at more extreme positions in other factors.

KenQ results supported this close reading. Z-scores and tests of distinguishing and consensus statements showed which items set one factor apart from the others and which items represented views shared across factors (see Supplementary Table S1). During interpretation, we checked these outputs against the visual pattern in the composite Q-sort grids. We used distinguishing statements to specify what defined each perspective, and we used consensus statements to describe areas of common ground within the participant group.

Interpretation also involved systematic cross-factor comparison and narrative accounts describing each factor’s core commitments, rejected positions, and internal tensions where necessary. For each factor, we compared the placement of each statement with its placement in the other arrays to identify where viewpoints converged and where they diverged. Throughout the narrative accounts, we referenced statement numbers and scores in parentheses to make clear which parts of the arrays supported each claim. After completing this whole-array interpretation, we assigned labels to the retained factors. These labels are meant to be as descriptive of the full attitude as possible and are interpretive shorthand for whole Q-sort configurations summarizing the main pattern of meaning within a factor array.

## 3. Results

### 3.1. Factor 1:“Faith-Integrated Institutional Trust”

Factor 1 had an eigenvalue of 11.77 and explained 39% of the study variance, making it the most prominent viewpoint in the participant set (Table 1). Twelve participants’ Q-sorts defined this factor. The group included 8 male and 4 female participants. Participants represented a range of academic backgrounds, including business (n = 5), STEM fields (n = 2), social sciences and education (n = 4), and arts and humanities (n = 1). Eight participants identified as Republican and four as Democrat (Table 2). Participants associated with this factor have broad trust in scientific evidence, public health institutions, and faith authorities. They frame vaccination as an ethical and communal practice that aligns with both personal belief and public responsibility. This viewpoint rejects individualist and conspiratorial rationales for refusal and emphasizes data-based, discussion-oriented education as the most effective way to sustain vaccine acceptance (Figure 3).

The Factor 1 viewpoint is unique in the way participants integrate their religious faith and scientific reasoning to shape vaccine attitudes. The strongest affirmation within this viewpoint is deep respect for science and a conviction that religious belief and scientific evidence are compatible. This is evident in statement 5, “I believe that my faith teaches me to respect science, including the science behind vaccines” (+5), and statement 1, “The best way to teach vaccine-related issues is by showing the data and addressing the controversies surrounding them” (+5). For these individuals, faith does not oppose vaccination; rather, it supports engagement with scientific and public health guidance.

This worldview is further seen in the high value placed on official information sources and institutional trust. Statement 30 (“I am confident that vaccines are thoroughly tested before being approved” [+4]) and 32 (“I trust information about vaccines from official health organizations, such as the Centers for Disease Control and Prevention (CDC) or the World Health Organization (WHO)” [+3]) are ranked much higher than in other factors, indicating confidence in vaccine safety and the transparency of side effect disclosure. Likewise, family and religious leadership strongly influence decisions; statement 26 (“My religious leaders’ opinions on vaccination matter to me” [+4]) and statement 19 (“My family’s opinions strongly influence my vaccination choices” [+1]) are both positive, further linking communal and faith contexts to acceptance.

Protection of vulnerable groups and communal responsibility are recurring themes. Participants feel a duty to vaccinate in order to help those who cannot be vaccinated (statement 14: “It is my responsibility to vaccinate to protect those who cannot do so themselves” [+2]), and they see vaccination as a form of service and care that aligns with their ethical, religious, and communal values (statement 25: “I see vaccination as an act of service to others, in line with my religious values” [+2]). The cultural value placed on protecting children is also pronounced (statement 7: “My cultural values place a high priority on protecting children, which includes vaccinating them” [+3]), and such commitments are viewed as central to sound public health practice (statement 13: “Vaccines are a key part of public health strategies to fight epidemics” [+3]).

Factor 1 individuals notably reject skepticism and narratives of vaccine refusal. They strongly disagree with statements such as 24 (“I believe that God will protect me from illness, so vaccination is unnecessary” [−5]) and 4 (“I have concerns about vaccines because they conflict with my religious beliefs” [−5]). Ideological resistance to mandates (statement 27: “I believe vaccine mandates infringe on personal freedoms” [−4]) and suspicion of scientists or profit motives (statement 31: “Scientists do not have the public’s best interests in mind regarding vaccines” [−4]; statement 34: “I believe vaccines are promoted for profit, not for health” [−1]) are also discounted, indicating a strong preference for expert and institutional sources over conspiracy-driven reasoning or politicized narratives.

Statements relating to individualism, misinformation, and hesitation from negative experiences received lower rankings in this factor than in others, indicating that trust is placed in collective expertise and observable benefits over isolated, personal, or ideological claims. For example, statement 15 (“I perceive vaccination as a personal choice rather than a public health responsibility” [−2]), statement 16 (“Misinformation about vaccines prevents me from getting vaccinated” [−3]), and statement 12 (“My hesitation about vaccines comes from my own experiences with illness” [−3]) are all ranked lower. Even statements placed at the middle of the distribution, such as statement 17 (“Learning new information and correcting misconceptions about vaccines is important” [0]) still suggest openness to discussion and learning rather than expressing doubt or disengagement.

### 3.2. Factor 2: “Skeptical Autonomy and Institutional Distrust”

Factor 2 had an eigenvalue of 4.07 and explained 14% of the study variance, making it the second most prominent viewpoint in the participant set (Table 1). Seven participants’ Q-sorts defined this factor. The group included 6 male participants and 1 female participant. Participants represented business (n = 3), STEM fields (n = 3), and arts and humanities (n = 1). All seven participants identified as Republican (Table 2). Factor 2 represents a viewpoint marked by institutional mistrust, reliance on personal experience and trusted anecdotes, and a strong emphasis on individual autonomy. This perspective is not defined by opposition to science or faith. Participants who hold this viewpoint are uncertain about credible information sources; they prefer to make personal judgments rather than follow official guidance. They believe that vaccination should be a personal choice rather than a collective responsibility (Figure 4).

Participants associated with Factor 2 express skepticism toward official and institutional sources of vaccine information. The strongest disagreement is reserved for trust in government health agencies (statement 28: “I trust government health agencies to provide accurate information” [−5]) and official organizations (statement 32: “I trust information about vaccines from official health organizations” [−5]), as well as for confidence in vaccine testing (statement 30: “I am confident that vaccines are thoroughly tested before they are approved for use” [−4]) and the value of learning new information (statement 17: “Learning new information and clarifying misconceptions about vaccines is important to me” [−4]). This mistrust is further confirmed in the highest agreement with statement 36 (“I find it hard to know which vaccine information sources to trust” [+5]), which stands out as a defining feature of this viewpoint (Figure 5).

Despite this skepticism, Factor 2 participants still affirm that their faith teaches respect for science and that vaccination aligns with their beliefs (statement 5: “I believe that my faith teaches me to respect science, including the science behind vaccines” [+5]), suggesting that their doubts are not rooted in religious opposition to science, but rather in a lack of confidence in the institutions that deliver vaccine information. This is further supported by the relatively high ranking of parental influence (statement 3: “My parents influence my vaccination decisions” [+4]) and the belief that vaccine mandates infringe on personal freedom (statement 27: “I believe vaccine mandates infringe on personal freedom” [+4]). This indicates that personal networks and autonomy are valued over institutional authority.

Personal experiences and stories play a significant role in shaping their attitudes. Statements such as statement 12 (“My hesitation about vaccines comes from my own experiences” [+3]) and statement 18 (“I am more hesitant about vaccines because of negative stories I’ve heard from people I trust” [+2]) are ranked higher than in other factors, highlighting the importance of anecdotal evidence and trusted personal sources. This group is also more likely to see vaccination as a personal choice rather than a public health responsibility (statement 15: [+3]), and to believe that health is a personal decision, not one for government regulation (statement 9: [+2]).

Factor 2 participants are more open to the idea that natural immunity is better than vaccine-acquired immunity (statement 35: [+1]) and that vaccines may be promoted for profit (statement 34: [+1]), further confirming their wariness of mainstream narratives. They are also more likely to agree that social media has shaped their views (statement 22: [0]) and that scientists may not have the public’s best interests in mind (statement 31: [0]), compared to other factors.

They are less likely to be influenced by family, friends, or community expectations (statements 8, 19, and 20: [0 to −2]), and they do not see vaccination as an act of service or a responsibility to others (statements 25, 14: [−2 to −1]). They also express less confidence in the safety and transparency of vaccines, disagreeing that side effects are fully disclosed (statement 33: [−3]) or that vaccines are thoroughly tested (statement 30: [−4]).

### 3.3. Factor 3: “Pragmatic Autonomy and Science Confidence”

Factor 3 had an eigenvalue of 1.85 and explained 6% of the study variance (Table 1). Ten participants’ Q-sorts defined this factor. The group included 9 male participants and 1 female participant. Participants represented STEM fields (n = 6), business (n = 2), social sciences and education (n = 1), and arts and humanities (n = 1). Nine participants identified as Republican and one as Democrat (Table 2). The Factor 3 viewpoint is a pro-vaccine viewpoint based on trust in science and official information, coupled with a clear preference for personal autonomy and family influence. Participants associated with this factor resist religious, ideological, and social pressures and prefer practical, transparent communication. Within this factor, vaccination is treated as both a public health good and an individual decision that should be supported through evidence and open discussion.

Factor 3 is defined by a pragmatic, science-affirming, and autonomy-oriented approach to vaccination. Participants who loaded on this factor strongly agreed with the view that “Vaccines are a key part of public health strategies to prevent disease” (statement 13, [+5]) and “The best way to teach vaccine-related issues is by showing the data and addressing concerns directly” (statement 1, [+5]). This group values clear, practical communication and sees vaccination as a cornerstone of public health, best promoted through transparent, evidence-based dialogue.

Trust in science and official sources is evident, but it is paired with a sense of personal agency. Statements like “I am confident that vaccines are thoroughly tested before they are approved for use” (statement 30, [+3]) and “I trust information about vaccines from official health organizations” (statement 32, [+3]) are ranked highly, as is trust in government health agencies (statement 28, [+2]). Yet, this trust does not mean that participants simply defer to official sources. Their vaccination views are also informed by personal judgment and family influence. “My parents influence my vaccination decisions” (statement 3, [+4]) and “My family’s opinions strongly influence my vaccination choices” (statement 19, [+1]) are both positive, indicating that close relationships play a meaningful role in shaping vaccine attitudes.

Personal autonomy is a strong feature of this viewpoint. “I perceive vaccination as a personal choice rather than a public health responsibility” (statement 15, [+3]) and “I believe that health is a personal decision, and I don’t think the government should be involved” (statement 9, [+2]), both indicate a preference for individual decision-making over collective mandates. This is further supported by moderate agreement with “I believe vaccine mandates infringe on personal freedoms” (statement 27, [+2]) and “I am more likely to trust vaccination information from sources like the CDC” (statement 29, [+2]), suggesting that while official sources are trusted, the ultimate decision is seen as personal.

Factor 3 participants are resistant to external pressures and skeptical of non-scientific influences. They strongly disagree that “God will protect me from illness, so vaccination is unnecessary” (statement 24, [−5]) and “I feel judged by others for their vaccination choices” (statement 23, [−5]). They reject the idea that social media (statement 22, [−4]) or religious concerns (statement 4, [−3]) should shape their vaccine views. They also disagree that scientists are untrustworthy (statement 31, [−3]) or that vaccine concerns should be based on negative stories or misinformation (statements 18 and 16: [−2] and [0]).

Middle-ranked items (0 to +1) show a balanced, evidence-oriented stance: participants neither find it hard to know which sources to trust (statement 36, [0]), nor do they strongly prefer natural immunity (statement 35, [0]). They are open to learning and clarifying misconceptions (statement 17, [0]), and do not report knowing anyone with serious vaccine side effects (statement 11, [+1]).

## 4. Discussion

This study identified three viewpoints on vaccination among students: Faith-Integrated Institutional Trust, Skeptical Autonomy and Institutional Distrust, and Pragmatic Autonomy and Science Confidence. These labels should be read as concise descriptions of the full Q-sort patterns, not as separate categories or fixed combinations of traits. Each label summarizes the main meaning of a factor array and represents a detailed description of viewpoints that emerged from this participant set and factor solution. Rather than discussing each factor in isolation, we organize the discussion around the following two central themes that cut across the viewpoints and allow clearer comparison of both shared concerns and important differences: (i) identity, trust, and the integration of faith and science, and (ii) personal autonomy versus collective responsibility in vaccination.

### 4.1. Identity, Trust, and the Integration of Faith with Science

A central theme across the three viewpoints identified in this study is the difference in the degree of trust in institutional science and how that trust intersects with participants’ religious and political identities. In our sample, a religiously homogeneous but demographically varied group in terms of academic background, gender, and political affiliation, we found that faith and science were not inherently in conflict. In fact, participants associated with all three viewpoints agree that their religious faith encourages respect for scientific knowledge, supporting previous research that faith and science are compatible [34]. However, they differ significantly in who and what they trust when it comes to making vaccination decisions. We explain these differences through the framework of identity-protective cognition (IPC). IPC refers to the tendency for individuals to respond to scientific information in ways that protect their cultural identity and social belonging [61]. When beliefs about a contested societal issue become linked to group identities, people often process information in ways that reinforce their group’s position and signal loyalty to it [62,63,64].

Factor 1, which we labeled “Faith-Integrated Institutional Trust”, represents a balanced integration of religious faith with confidence in science and public institutions. For students aligned with this factor, there is no contradiction between being a faithful member of a religious community and trusting vaccines. These students view scientific evidence as supporting their values. As a result, vaccine science appears faith-consistent, and data-centered teaching that directly addresses controversy feels identity-safe. This interpretation aligns with evidence that perceived support from religious leaders and family networks, together with higher trust in institutions and government health guidance, is associated with more positive vaccine attitudes and stronger willingness to vaccinate among religious individuals [32,34].

Students aligned with this factor trust the scientific community and government health agencies. They also view their religious identity as supportive of pro-health attitudes. Because their faith community and its leaders have supported vaccination [65], vaccine acceptance appears identity-consistent for these students. This alignment helps explain their rejection of anti-establishment and conspiracy-based claims. For example, they strongly disagreed with statements such as “God will protect me from illness, so vaccination is unnecessary” and “scientists do not have the public’s best interests in mind regarding vaccines.” For these participants, such claims conflict with both scientific reasoning and their faith’s emphasis on caring for health. Demographically, this viewpoint spanned various majors and included both conservative and liberal students, suggesting that shared religious values and education may bridge typical political divides. In a faith-based university setting, Viewpoint 1 indicates that a pro-vaccine stance can be framed as an expression of both faith and scientific reasoning. This challenges the broader assumption that religiosity inherently breeds vaccine skepticism [66]. Broader vaccine attitude research also shows that faith-based organizations can support vaccine confidence and uptake when vaccination is framed through trusted community relationships, public health service, and faith-consistent health values [67].

We labeled Factor 2 as “Skeptical Autonomy and Institutional Distrust.” Students aligned with this factor struggle with whom to trust. Despite sharing the same religious background as students in the other factors, they express strong skepticism toward scientific and public health authorities. In line with identity-protective cognition theory, political and cultural identity appears to shape this viewpoint. All participants defining this factor identified as politically conservative or Republican, and many came from business or technical fields. This is consistent with prior evidence that conservative ideologies can align with narratives that value self-reliance and wariness of government overreach [68,69]. For these students, accepting official public health messaging may feel like a threat to their identity as independent decision-makers and skeptics of government authority. They protect a worldview that prioritizes personal liberty and distrusts large institutions by questioning the credibility and motives of scientific and public health authorities.

Compared to other factors, Factor 2 relies more on personal stories and experiences. Trust is localized, centered on family and community anecdotes rather than extended to scientific leaders or federal agencies. This group’s relationship with faith is also somewhat localized. Although students aligned with this factor do not use religion as a reason to reject vaccines, they place personal freedom at the center of their views. They view vaccine mandates as an infringement on freedom. Thus, even though their church leaders have generally encouraged vaccination, these students’ broader identity places individual agency first. This suggests that their skepticism reflects uncertainty and distrust rather than absolute denial, since they do not claim that vaccines categorically do not work. Instead, they question the transparency and motives of those promoting vaccines, including whether side effects are fully disclosed or whether profit drives vaccine campaigns. Viewpoint 2 shows that even in a highly religious campus context, where the faith tradition does not reject science, some students may express vaccine hesitancy tied to distrust and identity-driven autonomy, similar to broader societal patterns of vaccine hesitancy.

Factor 3, which we labeled “Pragmatic Autonomy and Science Confidence,” sits between Factors 1 and 2 in a distinct way. Like Viewpoint 1, students aligned with this factor are generally pro-vaccine and confident in scientific evidence. Yet, like Viewpoint 2, they prioritize personal autonomy in health decisions. Students in this group show strong trust in science, but that trust does not translate into support for mandates or a strong view of vaccination as a moral obligation. They distinguished themselves by agreeing, more moderately, that “vaccine mandates infringe on personal freedom”. In other words, they accept the efficacy and public health value of vaccines while maintaining that the decision to vaccinate should remain with the individual.

This pragmatic yet autonomy-centered outlook appears consistent with a cultural identity that values both scientific education and personal responsibility. Indeed, nine out of ten defining participants for this factor were male and politically conservative, and many came from engineering or business disciplines. These backgrounds may support a mindset that values analytic reasoning, self-reliance, and individual decision-making. This helps explain how students in this viewpoint can trust scientific evidence while still resisting top-down directives. From an identity-protective cognition standpoint, Viewpoint 3 suggests that these students do not see scientific consensus as threatening to their identity. Pro-science views may be part of that identity, especially for students in technical fields. At the same time, autonomy remains central to how they understand health decisions. Thus, they balance acceptance of vaccines with reservations about compulsory measures.

Comparing these viewpoints through the theme of trust and identity integration expands our understanding of students’ underlying orientations toward vaccines and vaccination. First, religious faith itself is not the main source of division in this study, since none of the viewpoints frame vaccine hesitancy as a matter of doctrinal conflict. The findings show that both highly trusting and more skeptical vaccine attitudes can exist within a single faith community. These differences appear to be shaped by family influence and other dimensions of identity, including political worldview and personal experience. Second, institutional trust emerges as a key differentiator across the viewpoints. Viewpoint 1 trusts broadly, including health agencies, religious leaders, and scientific data. Viewpoint 2 trusts narrowly, preferring familiar people and personal networks over institutions. Viewpoint 3 trusts selectively, accepting scientific institutions while resisting the idea that they should dictate personal health decisions. The patterns identified in this study also appear in broader public discourse about vaccination. Prior research shows that higher trust in science and health authorities is associated with greater vaccine uptake [31,32,70,71,72,73,74], while anti-establishment and libertarian values are linked to greater vaccine skepticism and resistance to vaccine mandates [75,76,77,78]. These broader patterns mirror the differences observed across our factors. This study extends that literature by showing how trust orientations are experienced as holistic viewpoints and how they are tied to students’ sense of identity, belonging, and personal agency.

In post-sort interviews, students described their viewpoints in ways that closely matched the factor interpretations. A student associated with Viewpoint 1 framed faith and science as mutually reinforcing, stating, “My faith requires me to believe in science” and “I see science as a way we understand God and how He made the world.” This same student expressed confidence in scientific authority, explaining, “Yes, I trust scientific institutions. My parents trust them, so I grew up with that trust, and I did not question it much.” A student associated with Viewpoint 3 also expressed trust in vaccination and official health information, but in a way that was less institutionally grounded and more personally pragmatic. This student described vaccination as “a blessing from God” and said, “I feel like I do [trust official health information]. I feel like people in charge of health try to help people.” These interview comments support the factor interpretations and illustrate how identity-protective cognition operates across the viewpoints. Each group appears to accept information that fits its primary identity narrative while remaining cautious toward information that appears to threaten that narrative.

### 4.2. Personal Autonomy Versus Collective Responsibility in Vaccination

A second theme across the viewpoints is the tension between personal autonomy and collective responsibility in vaccination attitudes. A recent BYU influenza study found that political affiliation predicted influenza vaccination. The same dataset also showed a motivational shift from personal safety in 2007 to concern for public health in 2023, suggesting that students increasingly frame vaccination as a public good rather than only as a personal health decision [79]. Our study supports this pattern in one key way: all three viewpoints acknowledge the public health benefits of vaccination. What our study adds is a clearer explanation of how students from different political and identity backgrounds vary in the weight they give to communal duty versus individual choice.

The Health Belief Model (HBM) and Psychological Reactance Theory help interpret the differences across these viewpoints. The HBM proposes that health behaviors are shaped by beliefs about susceptibility to a health threat, the seriousness of that threat, the benefits of preventive action, perceived barriers, cues to action, and confidence in one’s ability to perform the behavior [80,81]. This framework helps explain how students weigh vaccine risks, benefits, and barriers. Psychological Reactance Theory adds another layer by explaining how people respond when they feel their freedom to choose is threatened. According to this theory, people may view certain behavioral choices as basic freedoms, and when those freedoms are restricted or threatened, they experience a motivational drive to restore them [82].

Factor 1 stands out for its pronounced sense of collective responsibility, informed by both ethical and religious values. This perspective aligns with communitarian values often emphasized in public health [83] and with faith teachings about health promotion and disease prevention [84]. Notably, all individuals loading on this factor were members of a faith community that places strong emphasis on service, mutual support, and care for others [85]. This is in line with the HBM’s concepts of perceived benefits and cues to action [81]. In this viewpoint, the benefits of vaccination are understood as both personal and social, involving protection of one’s own health as well as care for others and fulfillment of a moral duty. For these students, cues to action may include messages from trusted religious leaders that frame vaccination as stewardship or love for the community, as well as observations of family members and peers who prioritize public health. Their vaccination decisions are grounded in moral concern for the welfare of others and group well-being. As a result, students aligned with Factor 1 are more accepting of measures that frame vaccination as a social norm or shared responsibility. They do not view mandates primarily as violations of personal freedom because they understand public health as a collective obligation. This finding challenges the assumption that conservative or libertarian orientations are always defined by personal independence and limited obligation to others. Although Factor 1 was composed mostly of conservative students, they responded positively to messages centered on collective duty, protection of others, and shared responsibility.

Factors 2 and 3, on the other hand, lean strongly toward personal autonomy, although for different reasons. Factor 2 frames autonomy in more oppositional terms. Students aligned with this factor view public health regulations and one-size-fits-all messaging with suspicion, often as threats to liberty and personal control over health decisions. They express the view that vaccination should remain primarily an individual decision rather than a shared public obligation, and they believe that the government should not dictate health behaviors. From the perspective of Psychological Reactance Theory, strong emphasis on mandates and standardized public health messaging may evoke a sense of threatened freedom among these students. This perceived threat can produce reactance and motivate them to defend personal liberty, even if doing so may increase collective risk.

Although perceived severity and perceived benefits help explain why people adopt preventive health behaviors, the Health Belief Model also treats perceived barriers as central to decision-making. In Factor 2, distrust and concerns about personal freedom appear as barriers that outweigh perceived benefits [81]. Even when students recognize that vaccines may work, they may give less weight to the severity of the public health threat or the benefits to others than to personal freedom and self-determination. This finding suggests that, within this faith-based university context, vaccine skepticism can emerge even when religion itself is not used to reject science. For students aligned with Factor 2, the main barriers appear to be mistrust and concerns about personal autonomy, rather than complacency or lack of information.

For Factor 3, the tension between personal autonomy and collective responsibility does not appear as a rejection of vaccination or doubt about its scientific value. It appears that students aligned with this factor recognize vaccination as socially beneficial while still locating the final decision within the individual. One important finding is that these students hold a conditional view of collective responsibility. They strongly endorsed the idea that vaccines are a key part of public health strategies to fight epidemics. However, statements that framed vaccination as an act of service to others or as a direct responsibility to protect those who cannot protect themselves received much weaker support. This suggests that Factor 3 accepts the collective value of vaccination during a major public health crisis but does not strongly frame vaccination as a moral duty owed to others outside such a crisis. Interview data supported this interpretation. A participant associated with this factor stated, “I feel like everybody should still have a choice. Public health can help persuade, but it still comes down to choice.” This same student also acknowledged that “in a pandemic, vaccination becomes more of a responsibility to help maintain control of the virus.” Viewpoint 3 therefore shows an important variation within pro-vaccine attitudes: some individuals may trust vaccine science and recognize public health benefits while still preferring vaccination to remain a matter of individual choice rather than collective obligation.

Comparing across these viewpoints shows a continuum from communally oriented trust to individually oriented skepticism. Although Q methodology does not estimate how common each viewpoint is in the larger population, the three factors identified in this study show partial conceptual overlap with the 5C model of vaccine attitudes, which describes five psychological antecedents of vaccination: confidence, complacency, constraints, calculation, and collective responsibility [86]. For instance, the institutional trust and communal responsibility expressed in Factor 1 resemble the 5C dimensions of confidence and collective responsibility. The distrust, uncertainty, and autonomy concerns expressed in Factor 2 indicate lower confidence and higher calculation, as students carefully question which sources to trust before accepting vaccine information. Factor 3 shows high confidence in vaccine science but weaker collective responsibility, since students accept vaccination as useful while still framing the final decision as a matter of personal choice.

### 4.3. Implications for Classroom Communication

In this study, the value of these viewpoints lies in their capacity to guide communication and pedagogy within this specific context, with potential relevance to similar university settings. For students who hold Factor 1 viewpoints, instruction that links vaccination to religious values such as stewardship, care for the vulnerable, and respect for truth may align with their existing narratives. References to faith leaders who affirm science, together with clear presentation of evidence on vaccine risks and benefits, may also speak directly to their integrated trust in religious and scientific authority [34]. For students with a Factor 3 viewpoint, transparent and analytic teaching may have greater influence than additional appeals to authority. These students seek rational justification and room to reach their own conclusions. Therefore, classroom strategies that highlight methods, uncertainty, and tradeoffs, while still presenting clear evidence, may support their sense of agency and reinforce pro-vaccine reasoning.

Students with a Factor 2 viewpoint, however, require a different approach. For these students, relational trust and open dialogue matter more than repeated exposure to official guidance. Direct calls to obey government recommendations or accept agency claims at face value may deepen mistrust. Instructors should instead address concerns about profit, side effects, and past communication failures directly, using primary data and transparent discussion of how vaccine safety monitoring works. Framing vaccination as an informed choice before God and community, rather than as a test of loyalty to government, may better align with the ethic of agency that runs through this viewpoint. For these students, careful dialogue and acknowledgment of uncertainty are at least as important as factual content.

In a study where BYU college students interviewed family or community members who had experienced a vaccine-preventable disease, vaccine-hesitant students shifted toward more pro-vaccine attitudes after the interviews [36]. Other studies in highly religious Utah student and community settings suggest that openness to vaccine guidance is shaped by perceived in-group status and shared ideology, and they recommend pro-vaccine messages delivered by identifiable community members [35]. Building on these studies and the present findings, we conclude that dialogue-based and faith-sensitive pedagogy may influence vaccine attitudes across all three viewpoints. This approach is especially relevant because all three viewpoints valued opportunities to ask questions and instruction that acknowledges complexity, uncertainty, and controversy. In practice, this may involve classroom discussions centered on trusted in-group narratives about the consequences of vaccine-preventable diseases before students evaluate safety claims and institutional guidance. We therefore recommend that instructors invite students to examine evidence together, compare sources, and reflect on how faith and science can inform one another.

### 4.4. Implications for Public Health Messaging

Comparing the autonomy-versus-collective responsibility theme across viewpoints shows why public health messaging must be adaptive. For students aligned with Viewpoint 1, messages focused on solidarity, communal benefit, and faith-based values may reinforce their existing motivations. These students respond not only to data, but also to explanations of how those data connect to protecting loved ones and living their values. For the more autonomy-driven Viewpoint 2 and Viewpoint 3 groups, a different communication strategy is needed. Strong appeals to duty or authority may backfire if they are perceived as coercive. Communication should acknowledge autonomy while presenting evidence in a transparent and non-coercive way. For example, in a university classroom or campus health campaign, messages could emphasize that students are being given clear evidence about vaccine safety and disease risk so they can make informed choices for themselves and their community. This approach respects individual agency while still presenting a strong case for vaccination. Peer-led discussion forums and testimonials may also be useful, especially for students aligned with Viewpoint 2, who place greater trust in familiar people and personal networks. Policy framing can also be adjusted. Rather than presenting vaccination only as a requirement, institutions could frame it as an informed choice with community benefits. This may be more acceptable to students aligned with Viewpoint 3, who already recognize the benefits of vaccination, and may reduce resistance among students aligned with Viewpoint 2 by avoiding language that makes vaccination feel like a test of loyalty to government authority.

### 4.5. Theoretical and Practical Implications

Our findings support the idea that vaccine attitudes should not be analyzed in a one-dimensional way. Such analysis should account for personal beliefs, identity, and perceived norms. The Health Belief Model, Psychological Reactance Theory, and identity-protective cognition helped explain our data, but no single framework fully accounted for the pattern. HBM helps explain the cognitive dimensions of vaccine decision-making. However, HBM alone cannot fully explain why some participants distrusted government information so deeply or placed such strong value on freedom. This is where cultural identity and personal values fill gaps left by purely cognitive models of health behavior. Identity-protective cognition and psychological reactance help explain those gaps by showing how people may resist information or policies they perceive as threatening to their identity or autonomy.

Our study provides a refined, ground-level view of this: individuals with a strong communal/religious identity (Factor 1) embraced pro-vaccine information because it was in line with their group’s values (their church community encouraging care for others and respect for truth), whereas those whose identity emphasized individual liberty or skepticism (Factors 2 and 3) filtered the information differently, accepting the science only as far as it did not force them to betray those core values. This suggests that integrating cultural cognition perspectives with health behavior models could greatly enhance our understanding of vaccine attitudes and behaviors. In practical terms, interventions that resonate with a group’s identity, whether that identity is rooted in faith, political ideology, or professional/educational values, are likely to be more effective. For example, framing pro-vaccine messages in terms of conservative values like freedom (e.g., “vaccination as the quickest way to protect our freedoms by ending the pandemic”) or faith values (“loving thy neighbor by getting vaccinated”) can be seen as attempts to reduce the identity threat and make the health action identity consistent.

It is, however, important to note the context-specific and exploratory nature of these findings. Q-methodology is designed to map the variety of subjective viewpoints within a particular group, not to measure how many people in the broader population hold each view. Therefore, the demographic patterns reported in Table 2 provide useful context for interpreting the factor arrays, but they should not be treated as evidence that gender or political affiliation strictly predicts viewpoint membership. Our study does not claim that all college students or all BYU students fall into these three categories. These viewpoints point to key patterns of thought that exist in our student sample and may be present in other settings to varying degrees. BYU, as a private religious university, provided a unique context where all participants shared a common faith background and a high level of education. This commonality may have muted some divides (notably, none of our participants upheld an overtly religious anti-vaccine ideology), while highlighting others (such as political and personal autonomy differences).

### 4.6. Limitations

The following limitations should be noted. First, the final analytic sample included more male than female participants after data cleaning and quality screening. Although demographic characteristics were reported descriptively rather than used to predict factor membership, this imbalance limits our ability to examine whether similar viewpoints would emerge in a more gender-balanced sample. Second, the Q-sort was completed during a class activity, which may have created time constraints for some participants. Although the 37-statement Q-set was selected to reduce burden and fit within class time, some students may have needed more time to reflect on statement placement, especially given the forced distribution format. Third, the sample was not demographically heterogeneous in some respects, especially religious affiliation. All factor-defining participants reported affiliation with The Church of Jesus Christ of Latter-day Saints. This shared religious context was useful for examining variation within one faith-based university setting, but it limits the transferability of the findings to students from other religious traditions, secular institutions, or more religiously diverse campuses. Future studies should extend this work by repeating the Q-methodology procedure with students from other courses, campuses, religious traditions, and institutional contexts. They should also include more gender-balanced samples and link factor membership to survey-based measures of Health Belief Model constructs, identity-related variables, and reported vaccination behavior. Experimental studies that test communication strategies designed for each factor would provide an additional check on the practical value of these interpretations.

## 5. Conclusions

The viewpoints identified in this study suggest that effective vaccine communication should match the audience’s worldview. A single messaging approach is unlikely to work across groups because participants attach different meanings to vaccination. For some, vaccination is a collective duty, a faith commitment, and a show of trust in institutions, so messages that connect data to shared community values may strengthen acceptance. For others, vaccination is tied to independence and skepticism, so communication is more effective when it respects caution, addresses specific concerns directly, and supports informed choice. A third orientation combines confidence in scientific evidence with a strong preference for personal decision-making, which suggests the value of dialogue that is informative and noncoercive.

Public health educators and practitioners, including those working in universities or faith-based settings, can use these findings to guide more productive conversations. Messages are more likely to resonate when they acknowledge the identities and beliefs that shape vaccine attitudes, as reflected in both the Health Belief Model and identity-protective cognition. These results also indicate that faith and science, and personal freedom and public responsibility, need not be irreconcilable opposites.

## Figures and Tables

**Table 1:** Eigenvalues and explained variance by factor.

	Factor 1	Factor 2	Factor 3	Factor 4	Factor 5	Factor 6	Factor 7	Factor 8
Eigenvalues	11.769	4.066	1.848	1.496	1.390	1.253	1.198	0.943
% explained variance	39	14	6	5	5	4	4	3
cumulative % explained variance	39	53	59	64	69	73	77	80

**Table 2:** Demographic summary across three factors.

Demographic	Factor 1		Factor 2		Factor 3	
	Count	Weight	Count	Weight	Count	Weight
Gender						
Male	8	52.01	6	31.75	9	43.92
Female	4	18.86	1	6.24	1	4.19
Political Affiliation						
Republican	8	45.77	7	37.99	9	45.02
Democrat	4	25.10	-	-	1	2.89
Academic Major						
Business	5	33.95	4	22.68	-	-
STEM	3	10.99	3	9.07	3	18.14
Social Science/Edu	4	25.93	-	-	-	-
Arts (Violin performance)	-	-	1	6.24	-	-
Bus/Finance/Econ	-	-	-	-	3	13.32
Psychology	-	-	-	-	1	4.34
Religious Affiliation						
Church of Jesus Christ of Latter-day Saints	12	70.87	7	38.02	10	48.12

Note. Counts represent only Q-sorts that loaded significantly on a single factor. Weight refers to the summed contribution of defining Q-sorts within each demographic category to the construction of the factor array. Of the 30 Q-sorts included in the analysis, 29 loaded significantly on one of the three retained factors and are represented in this table. One Q-sort did not load significantly on any factor and was therefore not included in the factor-based demographic summary. Across the full set of 30 participants, 23 identified as male and 7 as female. When political affiliations were grouped, 24 participants were classified as Republican-aligned (including Republican, Conservative, and Lean Right) and 6 as Democrat-aligned (including Democrat, Liberal, and Liberal Democrat). All participants in this study reported affiliation with The Church of Jesus Christ of Latter-day Saints.

## Data Availability

Data will be made available on BYU Scholars Archive upon acceptance of the manuscript.

## Supplementary Materials

The following supporting information can be downloaded at: https://www.mdpi.com/article/10.3390/vaccines14060546/s1. Table S1: Statement rankings and standardized factor scores for the final 37-statement vaccine-attitudes Q-set across the three retained factors; Table S2: Post-sort interview protocol; Table S3: Coding framework used to review and balance the vaccine-attitudes concourse; Table S4: Final 37-statement Q-set mapped to coding domains and vaccine-attitude positions; Table S5: Selection rules used during concourse reduction.

## Abbreviations

The following abbreviations are used in this manuscript:

BYU	Brigham Young University
CDC	Centers for Disease Control and Prevention
COVID-19	Coronavirus Disease 2019
HBM	Health Belief Model
HPV	Human Papillomavirus
IPC	Identity-Protective Cognition
IRB	Institutional Review Board
P-set	Participant-set
PCA	Principal Component Analysis
STEM	Science, Technology, Engineering, and Mathematics
VPDs	Vaccine-Preventable Diseases
WHO	World Health Organization
