# Supplementary material for: Faith, Science, and Choice: Vaccine Attitudes Among Religious University Students
Source: Vaccines (Basel). 2026 Jun 20;14(6):546. doi: 10.3390/vaccines14060546 (PMC13307724; doi:10.3390/vaccines14060546)
Supplement: Supplementary file 1 [file vaccines-14-00546-s001.zip › vaccines-4332456-supplementary.pdf]

vaccines

MDPI

Faith, Science, and Choice: Vaccine Attitudes Among Religious University Students

Table S1: Statement rankings and standardized factor scores for the final 37-statement vaccine-attitudes Q-set across the three retained factors

Statement Number	Statement	Factor 1		Factor 2		Factor 3	
		Rank	Z-score	Rank	Z-score	Rank	Z-score
1	The best way to teach vaccine-related issues is by showing the data and addressing the controversies surrounding them	1	1.53	2	0.64	12	1.71
**2**	**My vaccine attitude is influenced by political ideologies**	**2**	**-0.79**	**29**	**-0.46**	**22**	**-0.31**
**3**	**My parents influence my vaccination decisions**	**3**	**0.96**	**10**	**1.34**	**4**	**1.39**
4	I have concerns about vaccines because they conflict with my religious beliefs about purity and bodily autonomy	4	-1.89	37	-0.8	29	-1.33
**5 ***	**I believe that my faith teaches me to respect science, including the science behind vaccines**	**5**	**1.67**	**1**	**1.58**	**2**	**1.58**
6	I believe that vaccinating children against Human Papillomavirus (HPV), a sexually transmitted infection, sends mixed messages about sexual activity	6	-0.44	21	0.44	16	-1.4
7	My cultural values place a high priority on protecting children, which includes vaccinating them	7	1.18	5	0.55	14	0.53
**8 ***	**I'm influenced by what most people in my community do and expect when it comes to vaccination**	**8**	**-0.49**	**22**	**-0.56**	**25**	**-0.31**
9	I believe that health is a personal decision, and I don't think the government should regulate it	9	-0.73	28	0.92	9	0.68
10	I have seen firsthand the effects of vaccine-preventable diseases, and that influences my decision to vaccinate	10	0.52	14	-0.48	23	-0.1
11	I don't know anyone who has experienced a serious side effect from a vaccine, so I don't have any problems getting vaccinated	11	0.05	18	-1.07	31	0.59
12	My hesitation about vaccines comes from my own experiences with illness	12	-1.08	33	1.03	7	-0.87
13	Vaccines are a key part of public health strategies to fight epidemics	13	1.17	6	0.91	10	1.76
14	It is my responsibility to vaccinate to protect those who cannot do so themselves	14	1.01	8	-0.61	26	-0.45
15	I perceive vaccination as a personal choice rather than a public health responsibility	15	-0.8	30	1.34	5	1.25
16	Misinformation about vaccines prevents me from getting vaccinated	16	-1.08	32	-0.28	20	-0.17
17	Learning new information and clarifying misconceptions about the COVID-19 vaccine encouraged me to get vaccinated	17	0.27	17	-1.28	34	-0.36
18	I am more hesitant about vaccines because of negative stories I've heard from people I trust	18	-0.54	23	0.99	8	-0.76
**19 ***	**My family's opinions strongly influence my vaccination choices**	**19**	**0.65**	**12**	**0.25**	**17**	**0.4**

20	My friends' attitudes toward vaccination affect my own views	20	-0.33	20	-1.01	30	-1.11
**21**	**My healthcare provider's recommendations are not important to me when deciding about vaccines**	**21**	**-1.02**	**31**	**-0.75**	**27**	**-1.36**
22	Social media has shaped my views on vaccination	22	-0.55	24	-0.08	18	-1.36
23	I feel judged by others for my vaccination choices	23	-0.72	27	-0.49	24	-1.6
24	I believe that God will protect me from illness, so vaccination is unnecessary	24	-1.78	36	-1.16	32	-1.6
25	I see vaccination as an act of service to others, in line with my religious values	25	1.01	9	-0.76	28	-0.64
26	My religious leaders' opinions on vaccination matter to me	26	1.42	3	1.08	6	-0.13
27	I believe vaccine mandates infringe on personal freedom	27	-1.18	34	1.55	3	0.4
28	I trust government health agencies to provide accurate information about vaccines	28	0.9	11	-1.58	36	0.85
29	I am more likely to trust vaccination information from sources that share my political views	29	0.35	16	-0.16	19	0.59
30	I am confident that vaccines are thoroughly tested before being approved	30	1.32	4	-1.51	35	1.2
31	Scientists do not have the public's best interests in mind regarding vaccines	31	-1.44	35	-0.34	21	-1.21
32	I trust information about vaccines from official health organizations, such as the Centers for Disease Control and Prevention (CDC) or the World Health Organization (WHO)	32	1.09	7	-1.63	37	1.04
33	I believe that vaccine side effects are fully disclosed to the public	33	0.49	15	-1.22	33	0.36
34	I believe vaccines are promoted for profit, not for health	34	-0.62	25	0.5	15	-0.54
35	I believe natural immunity is better than vaccine-acquired immunity	35	-0.7	26	0.55	13	0
36	I find it hard to know which vaccine information sources to trust	36	-0.04	19	1.76	1	0.24
**37 ***	**The best way to teach vaccine-related issues is by showing the data**	**37**	**0.59**	**13**	**0.8**	**11**	**0.98**

Z-score: standardized factor scores for each statement, computed as the weighted average of scores from defining Q-sorts for that factor.
Rank: position of each statement within a factor, ordered from 1 (strongest agreement) to 37 (strongest disagreement), used to build the factor arrays for interpretation.
Bolded statements are consensus items that do not distinguish between any pair of factors, meaning their scores do not differ significantly across the factor solution.
*Consensus statement significant at <0.05.

Q-Set

1. The best way to teach vaccine-related issues is by showing the data and addressing the controversies surrounding them.
2. My vaccine attitude is influenced by political ideologies.
3. My parents influence my vaccination decisions.
4. I have concerns about vaccines because they conflict with my religious beliefs about purity and bodily autonomy.
5. I believe that my faith teaches me to respect science, including the science behind vaccines.

6. I believe that vaccinating children against Human Papillomavirus (HPV), a sexually transmitted infection, sends mixed messages about sexual activity.
7. My cultural values place a high priority on protecting children, which includes vaccinating them.
8. I'm influenced by what most people in my community do and expect when it comes to vaccination.
9. I believe that health is a personal decision, and I don't think the government should regulate it.
10. I have seen firsthand the effects of vaccine-preventable diseases, and that influences my decision to vaccinate.
11. I don't know anyone who has experienced a serious side effect from a vaccine, so I don't have any problems getting vaccinated.
12. My hesitation about vaccines comes from my own experiences with illness.
13. Vaccines are a key part of public health strategies to fight epidemics.
14. It is my responsibility to vaccinate to protect those who cannot do so themselves.
15. I perceive vaccination as a personal choice rather than a public health responsibility.
16. Misinformation about vaccines prevents me from getting vaccinated.
17. Learning new information and clarifying misconceptions about the COVID-19 vaccine encouraged me to get vaccinated.
18. I am more hesitant about vaccines because of negative stories I've heard from people I trust.
19. My family's opinions strongly influence my vaccination choices.
20. My friends' attitudes toward vaccination affect my own views.
21. My healthcare provider's recommendations are not important to me when deciding about vaccines.
22. Social media has shaped my views on vaccination.
23. I feel judged by others for my vaccination choices.
24. I believe that God will protect me from illness, so vaccination is unnecessary.
25. I see vaccination as an act of service to others, in line with my religious values.
26. My religious leaders' opinions on vaccination matter to me.
27. I believe vaccine mandates infringe on personal freedom.
28. I trust government health agencies to provide accurate information about vaccines.
29. I am more likely to trust vaccination information from sources that share my political views.
30. I am confident that vaccines are thoroughly tested before being approved.
31. Scientists do not have the public's best interests in mind regarding vaccines.
32. I trust information about vaccines from official health organizations, such as the Centers for Disease Control and Prevention (CDC) or the World Health Organization (WHO).
33. I believe that vaccine side effects are fully disclosed to the public.
34. I believe vaccines are promoted for profit, not for health.
35. I believe natural immunity is better than vaccine-acquired immunity.
36. I find it hard to know which vaccine information sources to trust.
37. The best way to teach vaccine-related issues is by showing the data.

Pre-Sort Interview Protocol

1. How do you feel about vaccines in general?
2. What are your thoughts on vaccine effectiveness? Do you believe vaccines work as intended?

3. Do you have any concerns about vaccine safety? If so, what are they based on?
4. How much trust do you have in public health agencies (e.g., CDC, WHO) and pharmaceutical companies regarding vaccines?
5. How do personal experiences (either your own or those of people you know) influence your views on vaccines?
6. Do you believe religious or cultural beliefs play a role in vaccination decisions? If so, how?
7. How do family, friends, healthcare providers, and religious leaders influence your vaccination decisions?
8. What are your thoughts on vaccine mandates for schools or workplaces?
9. Do you think misinformation or distrust in the government impacts vaccine attitudes? Why or why not?
10. What do you think would encourage more people to get vaccinated?
11. How do you think vaccines should be discussed in classrooms? What would be the most effective way to teach it?
12. What do you think other people would say to these questions who may have a different opinion than you?

Table S2: Post-Sort Interview Protocol

**Factor 1: Faith-Integrated Institutional Trust**	
**Theme**	**Questions**
**1. Faith and Science Connection**	You strongly agreed with the statement, “My faith teaches me to respect science, including the science behind vaccines.”
	a. What experiences or religious teachings led you to this belief?
	b. How do you see faith and science supporting each other in vaccine decisions?
	You also agreed with the statement, “The best way to teach vaccine issues is by showing data and addressing controversies.”
	a. What kind of data do you think is most convincing?
	b. What controversies do you think matter most, and how should they be addressed?
	c. In a BYU classroom, how could vaccine controversies be discussed in a way that also allows room for gospel perspectives?
**2. Institutional Trust**	You placed high confidence in vaccine testing and information from government health agencies.
	a. What makes these sources credible to you?
	b. Are there any limits to your trust in these sources?
	c. How do you decide whether information feels trustworthy?
	If a church leader and a public health official disagreed about a vaccine, how would you respond?
**3. Moral and Communal Duty**	You agreed that vaccinating protects others and serves the community.
	a. In your view, how do vaccines protect other people?
	b. Do you see vaccination as part of your responsibility, as a member of the Church, to protect and serve your community?
	You also ranked the statement, “I see vaccination as an act of service to others, in line with my religious values,” highly.
	a. What do you think is the Church’s view of vaccination?

	b. Can you describe a time when being vaccinated felt like an ex pression of your val- ues?
**4. Rejecting Opposition**	You strongly disagreed with the statement, "God will protect me from illness, so vaccination is unnecessary."<br>a. Why do you disagree with this statement?<br>b. What is your view of divine intervention in times of illness?<br>You also disagreed with the statement, "I have concerns about vaccines because they conflict with my religious beliefs."<br>a. Do you believe vaccines are consistent with your religious be-liefs? Why?<br>b. Why do you think some religious individuals struggle with vaccines?
**5. Personal and Family Influence**	You ranked family influence as moderately important in shaping your views about vaccination.<br>a. Who in your family or community influences your vaccine decisions the most?<br>b. How do family discussions about vaccines compare with what you learn at school or at church?
**6. Reflection and Broader View**	Do you think people who share your faith but oppose vaccines misunderstand doctrine, science, or both?<br>Has your perspective changed at all since completing the Q-sort?<br>If you were creating a message about vaccination for your faith community, what would you want it to focus on?<br>What are your thoughts on the way I described this factor? Does "Faith-Integrated Institutional Trust" feel accurate to you, or would you describe your perspective differently?

Factor 2: Skeptical Autonomy and Institutional Distrust

**Theme**	**Questions**
**1. Distrust and Information Confusion**	You ranked the statement, "It is hard to know which vaccine information sources to trust," very highly.<br>a. What makes information about vaccines confusing or unreliable to you?<br>b. Have you found any sources that feel more trustworthy than others?<br>You disagreed with the statement, "I trust government health agencies to provide accurate information."<br>a. What experiences shaped your view of government or public health institutions?<br>b. What would those institutions need to do to earn your trust?
**2. Autonomy and Freedom**	You agreed with the statement, "Vaccine mandates infringe on personal freedom."<br>a. What personal freedoms do you feel are being infringed upon?<br>b. Where do you think the boundaries of personal freedom should lie in public health decisions such as vaccination?<br>c. Are there any situations in which a vaccine mandate would feel acceptable to you?<br>You also agreed with the statement, "Health is a personal decision, not one the government should regulate."<br>a. Why do you feel the government should not be involved in personal health decisions?<br>b. What risks do you see in government involvement in those decisions?

**3. Personal and Anecdotal Experience**	You placed weight on negative stories from people you trust and on your own experiences related to vaccines.<br>a. Can you recall a story or event that made you question vaccine safety?<br>b. How have these personal experiences influenced your decisions about vaccines?<br>c. How do you make vaccine decisions when personal experiences seem to conflict with mainstream scientific recommendations?
**4. Faith and Leadership**	You agreed that your faith teaches respect for science, yet you also showed distrust in official scientific institutions.<br>a. What evidence, teachings, or statements lead you to believe your faith teaches respect for science?<br>b. What causes you to respect science while distrusting certain scientific agencies?<br>c. Have any particular events contributed to that distrust?<br>d. Do your church leaders influence your vaccine decisions? If so, how?
**5. Communication and Messaging**	You agreed that "showing data and addressing controversies" is a good way to teach people about vaccines.<br>a. What kind of data would be most convincing to you?<br>b. What controversies should be addressed, and how should they be discussed?<br>c. What kinds of information do you find unconvincing or unhelpful?<br>If you were creating a message for students who share your concerns, what approach would you use to help them feel comfortable?
**6. Reflection**	Looking back at your Q-sort, do you think your caution comes more from information overload, personal experiences, values about freedom, or something else?<br>What would need to happen for you to feel more confident about vaccines?

Factor 3: Pragmatic Autonomy and Science Confidence

**Theme**	**Questions**
**1. Science and Data Confidence**	You strongly agreed with the statement, "Vaccines are a key part of public health strategies to fight epidemics."<br>a. What experiences or information make you confident that vaccines are effective in fighting epidemics?<br>You also ranked the statement, "The best way to teach vaccine-related issues is by showing data and addressing controversies," highly.<br>a. What type of data presentation feels most persuasive to you?<br>b. What do you think would persuade others who may be hesitant about vaccines?<br>c. How can educators discuss controversial vaccine topics effectively without alienating students?
**2. Personal Autonomy and Responsibility**	You agreed with the statement, "Vaccination is a personal choice rather than a public health responsibility."<br>a. What does personal choice mean to you in this context?<br>b. What does public health responsibility mean to you?<br>c. Are there situations in which public health concerns should take priority over individual choice?<br>You also agreed with the statement, "Health is a personal decision, and the government should not regulate it."

	a. Why do you think individuals are better positioned than the government to make these decisions?
	b. What risks do you see in allowing the government to regulate these decisions?
	c. How do you view policies that require vaccination for travel, work, or school?
**3. Family and Social Influence**	You gave some weight to family information in making decisions about vaccination.
	a. Does anyone else influence your views about vaccines? If so, who and why?
	b. How do you handle disagreement about vaccines within your family or friend group?
**4. Faith and Reason**	You disagreed with the statement, "God will protect me from illness, so vaccination is unnecessary."
	a. Why do you disagree with this statement?
	b. What is your view of divine intervention in times of illness?
	In your view, what does the Church teach about using medical advances such as vaccines?
	You also disagreed with the statement, "Vaccines conflict with religious beliefs about purity and bodily autonomy."
	a. Why do you think some people in your faith community see this as a conflict?
**5. Communication and Trust**	How do you think trust in official health information shapes people's vaccine decisions?
	What advice would you give professors or public health professionals who want to communicate effectively with students who think the way you do?
**6. Reflection**	How would you explain your view of vaccines and individual choice in relation to public health?
	a. Do you see any tension between personal autonomy and collective responsibility in public health?
	After completing your Q-sort, did you gain any new insights into your own views or the views of others about vaccination?

Table S3. Coding framework used to review and balance the vaccine-attitudes concourse

Code	Content domain	Domain description	Spectrum coverage	Final Q-set	Example retained item(s)	Example excluded or merged item	Reason for exclusion or revision
VE	Vaccine effectiveness and public health value	Statements about vaccine efficacy, public health value, herd immunity, disease prevention, and natural immunity comparisons.	Acceptance, confidence, uncertainty about efficacy	2	13. Vaccines are a key part of public health strategies to fight epidemics.<br>35. I believe natural immunity is better than vaccine-acquired immunity.	Vaccination is one of the greatest public health achievements of the 20th century.	Merged with broader public-health value items to avoid repetition.
SC	Safety concerns, testing, and side effects	Statements about vaccine testing, side effects, safety, and disclosure.	Confidence, safety concern, transparency concern	2	30. I am confident that vaccines are thoroughly tested before being approved.<br>33. I believe that vaccine side effects are fully disclosed to the public.	Vaccines can cause side effects, but they are usually minor and temporary.	Excluded because the final set used more direct safety-testing and side-effect disclosure statements.

Code	Content domain	Domain description	Spectrum coverage	Final Q-set	Example retained item(s)	Example excluded or merged item	Reason for exclusion or revision
SK	Skepticism about pharmaceutical or scientific motives	Statements questioning pharmaceutical, scientific, or profit motives.	Skepticism, distrust	1	34. I believe vaccines are promoted for profit, not for health.	Pharmaceutical companies prioritize profit over public health when it comes to vaccines.	Condensed into a shorter profit-motive statement.
GPH	Government role, regulation, and personal health decisions	Statements about government roles in health, regulation, and personal decision-making.	Autonomy, regulation concern	1	9. I believe that health is a personal decision, and I don't think the government should regulate it.	Government-provided healthcare is essential for ensuring everyone has access to vaccines.	Excluded because the study focus was vaccine attitudes rather than healthcare policy preferences.
PES	Personal economic status and vaccine access	Statements about cost, income, and economic access to vaccination.	Access concern	0	Not retained in final Q-set; reviewed during balancing.	Economic hardship can make it harder for some people to prioritize vaccines.	Excluded because access and cost were less central to the study context and overlapped with structural-barrier items.
PEV	Personal or vicarious experience with disease or vaccine effects	Statements about direct or indirect experience with vaccine-preventable disease or adverse effects.	Experience-based acceptance or hesitation	3	10. I have seen firsthand the effects of vaccine-preventable diseases, and that influences my decision to vaccinate.<br>11. I don't know anyone who has experienced a serious side effect from a vaccine, so I don't have any problems getting vaccinated.	Personal stories of children affected by vaccine-preventable diseases strongly encourage me to vaccinate.	Merged with firsthand and vicarious experience items.
VHA	Vaccine hesitancy, acceptance, and uncertainty	Statements describing uncertainty, acceptance, hesitation, refusal, or source difficulty.	Acceptance, ambivalence, hesitancy, refusal	1	36. I find it hard to know which vaccine information sources to trust.	I'm hesitant because of the potential side effects, even though the risks are generally low.	Represented through safety, uncertainty, and source-trust items to avoid overloading the set with hesitancy-framed statements.
RB	Religious and cultural beliefs	Statements about religious beliefs, faith-science compatibility, cultural values, purity, and bodily autonomy.	Faith-science acceptance, religious concern, religious refusal	5	4. I have concerns about vaccines because they conflict with my religious beliefs about purity and bodily autonomy.<br>5. I believe that my	My religious community generally supports vaccination, so I feel comfortable getting vaccinated.	Merged with faith-science and religious-leader influence items.

Code	Content domain	Domain description	Spectrum coverage	Final Q-set	Example retained item(s)	Example excluded or merged item	Reason for exclusion or revision
					faith teaches me to respect science, including the science behind vaccines.		
ALT	Altruism, community responsibility, and care for others	Statements about moral responsibility, service, care for vulnerable people, and collective responsibility.	Collective responsibility, moral acceptance, personal-choice tension	3	14. It is my responsibility to vaccinate to protect those who cannot do so themselves.<br>15. I perceive vaccination as a personal choice rather than a public health responsibility.	I believe getting vaccinated is a way to demonstrate compassion for those at risk.	Merged with responsibility and service-to-others statements.
SI	Social influences, family, peers, providers, and religious leaders	Statements about family, peers, community norms, healthcare providers, and religious leaders.	Family, peer, provider, community, and faith-leader influence	8	3. My parents influence my vaccination decisions.<br>8. I'm influenced by what most people in my community do and expect when it comes to vaccination.	My friends and family trust vaccines, which has influenced my own decision to vaccinate.	Separated into family, friends, parents, and religious-leader influence items.
VM	Support for or opposition to vaccine mandates	Statements supporting or opposing vaccine mandates and requirements.	Mandate support or opposition	1	27. I believe vaccine mandates infringe on personal freedom.	I support vaccine mandates to prevent the spread of infectious diseases in schools.	Balanced with a mandate-opposition item so the final set did not overrepresent mandate support.
TPH	Trust or distrust in public health agencies and scientific institutions	Statements about trust or distrust in government agencies, scientific institutions, CDC/WHO, and scientists.	Institutional trust or distrust	3	28. I trust government health agencies to provide accurate information about vaccines.<br>31. Scientists do not have the public's best interests in mind regarding vaccines.	I rely on public health agencies to provide clear and accurate vaccine information.	Condensed into trust in government health agencies and CDC/WHO statements.
VC	Vaccine communication, education, and interventions	Statements about communication, education, data use, controversy, and correcting misconceptions.	Evidence-centered communication, uncertainty reduction	3	1. The best way to teach vaccine-related issues is by showing the data and addressing the controversies surrounding them.<br>17. Learning new information and	Interventions will be most effective when incorporated with interactive communication training rather than just	Excluded because it described intervention design rather than a first-person vaccine attitude.

Code	Content domain	Domain description	Spectrum coverage	Final Q-set	Example retained item(s)	Example excluded or merged item	Reason for exclusion or revision
					clarifying misconceptions about the COVID-19 vaccine encouraged me to get vaccinated.	providing factual information.	
SLB	Structural and logistical barriers to vaccination	Statements about access barriers, time, transportation, reminders, and healthcare access.	Structural barriers	0	Not retained in final Q-set; reviewed during balancing.	I don't want to be vaccinated because of a lack of time.	Excluded because logistical barriers were less central to the faith, identity, and trust focus of the study.
NAP	Nonengagement, complacency, or apathy	Statements about complacency, lack of engagement, or low perceived relevance.	Complacency, disengagement	0	Not retained in final Q-set; reviewed during balancing.	Vaccines' success in reducing diseases has led to complacency.	Excluded because it was framed as a general claim rather than a self-referential statement.
MIE	Misinformation and information environment	Statements about misinformation, social media, information quality, and source confusion.	Misinformation, source uncertainty, social media influence	2	16. Misinformation about vaccines prevents me from getting vaccinated. 22. Social media has shaped my views on vaccination.	Misinformation and distrust in government institutions play a crucial role in vaccine hesitancy.	Revised into shorter self-referential misinformation and source-trust items.
PI	Political and ideological influences	Statements about political ideology, political leaders, and identity-aligned sources.	Political identity and source trust	2	2. My vaccine attitude is influenced by political ideologies. 29. I am more likely to trust vaccination information from sources that share my political views.	Political affiliation is associated with vaccine uptake, with Democrats being more likely to receive the COVID-19 vaccine compared to Republicans.	Excluded because it was a literature-summary statement rather than a sort-ready personal attitude.
PBF	Psychological and behavioral factors in vaccine decision-making	Statements about psychological mechanisms such as risk perception, fear, calculation, and identity-protective cognition.	Risk perception, fear, calculation, identity-protective cognition	0	Not retained in final Q-set; reviewed during balancing.	Addressing emotional and psychological barriers to vaccination is just as important as providing factual information.	Excluded because it described a theoretical mechanism rather than a first-person viewpoint.

Note. Final Q-set counts reflect the primary code assigned to each final statement. Some statements could reasonably fit more than one domain, but each was assigned one primary code for audit-trail clarity.

Table S4. Final 37-statement Q-set mapped to coding domains and vaccine-attitude positions

No.	Final Q-set statement	Primary code	Primary domain	Attitudinal position	Selection rationale
1	The best way to teach vaccine-related issues is by showing the data and addressing the controversies surrounding them.	VC	Vaccine communication, education, and interventions	Communication/education; evidence-centered acceptance	Retained to capture support for data-centered vaccine instruction with controversy addressed directly.
2	My vaccine attitude is influenced by political ideologies.	PI	Political and ideological influences	Political identity influence	Retained to capture political ideology as a possible source of vaccine-attitude formation.
3	My parents influence my vaccination decisions.	SI	Social influences, family, peers, providers, and religious leaders	Family influence	Retained to capture parental influence as a close-network source of vaccine decision-making.
4	I have concerns about vaccines because they conflict with my religious beliefs about purity and bodily autonomy.	RB	Religious and cultural beliefs	Religious concern or conflict	Retained to capture faith-based concerns around purity and bodily autonomy.
5	I believe that my faith teaches me to respect science, including the science behind vaccines.	RB	Religious and cultural beliefs	Faith-science compatibility and vaccine confidence	Retained to capture religious support for science and vaccination.
6	I believe that vaccinating children against Human Papillomavirus (HPV), a sexually transmitted infection, sends mixed messages about sexual activity.	RB	Religious and cultural beliefs	Religious or moral concern around HPV vaccination	Retained to capture sexuality-related concerns tied to HPV vaccination in religious contexts.
7	My cultural values place a high priority on protecting children, which includes vaccinating them.	RB	Religious and cultural beliefs	Cultural values and acceptance	Retained to capture cultural responsibility for child protection.
8	I'm influenced by what most people in my community do and expect when it comes to vaccination.	SI	Social influences, family, peers, providers, and religious leaders	Community norms	Retained to capture perceived social norms and community expectations.
9	I believe that health is a personal decision, and I don't think the government should regulate it.	GPH	Government role, regulation, and personal health decisions	Autonomy and government noninterference	Retained to capture health as a personal decision and concern about government regulation.
10	I have seen firsthand the effects of vaccine-preventable diseases, and that influences my decision to vaccinate.	PEV	Personal or vicarious experience with disease or vaccine effects	Disease experience and acceptance	Retained to capture firsthand experience with vaccine-preventable disease.
11	I don't know anyone who has experienced a serious side effect from a vaccine, so I don't have any problems getting vaccinated.	PEV	Personal or vicarious experience with disease or vaccine effects	Absence of adverse-event experience and confidence	Retained to capture lack of negative vaccine experience as a source of comfort.
12	My hesitation about vaccines comes from my own experiences with illness.	PEV	Personal or vicarious experience with disease or vaccine effects	Personal experience and hesitation	Retained to capture illness experience as a source of hesitation.
13	Vaccines are a key part of public health strategies to fight epidemics.	VE	Vaccine effectiveness and public health value	Vaccine confidence and public health value	Retained to capture strong acceptance of vaccination as epidemic control.

No.	Final Q-set statement	Primary code	Primary domain	Attitudinal position	Selection rationale
14	It is my responsibility to vaccinate to protect those who cannot do so themselves.	ALT	Altruism, community responsibility, and care for others	Collective responsibility and protection	Retained to capture moral responsibility to protect medically vulnerable people.
15	I perceive vaccination as a personal choice rather than a public health responsibility.	ALT	Altruism, community responsibility, and care for others	Autonomy versus public responsibility	Retained to capture vaccination as personal choice rather than public health duty.
16	Misinformation about vaccines prevents me from getting vaccinated.	MIE	Misinformation and information environment	Misinformation and hesitancy	Retained to capture misinformation as a barrier to vaccination.
17	Learning new information and clarifying misconceptions about the COVID-19 vaccine encouraged me to get vaccinated.	VC	Vaccine communication, education, and interventions	Learning, correction of misconceptions, and acceptance	Retained to capture education and clarification as factors affecting vaccine acceptance.
18	I am more hesitant about vaccines because of negative stories I've heard from people I trust.	SI	Social influences, family, peers, providers, and religious leaders	Trusted stories and hesitancy	Retained to capture negative trusted stories as a source of hesitation.
19	My family's opinions strongly influence my vaccination choices.	SI	Social influences, family, peers, providers, and religious leaders	Family influence	Retained to capture broader family influence beyond parents.
20	My friends' attitudes toward vaccination affect my own views.	SI	Social influences, family, peers, providers, and religious leaders	Peer influence	Retained to capture friends' attitudes as a social influence.
21	My healthcare provider's recommendations are not important to me when deciding about vaccines.	SI	Social influences, family, peers, providers, and religious leaders	Healthcare provider influence, low salience	Retained to capture resistance to professional recommendation as a decision source.
22	Social media has shaped my views on vaccination.	MIE	Misinformation and information environment	Social media influence	Retained to capture social media as part of the vaccine information environment.
23	I feel judged by others for my vaccination choices.	SI	Social influences, family, peers, providers, and religious leaders	Social judgment and vaccine identity	Retained to capture perceived judgment around vaccination choices.
24	I believe that God will protect me from illness, so vaccination is unnecessary.	RB	Religious and cultural beliefs	Religious refusal	Retained to capture an explicitly faith-based reason for rejecting vaccination.
25	I see vaccination as an act of service to others, in line with my religious values.	ALT	Altruism, community responsibility, and care for others	Religious service and collective care	Retained to capture vaccination as faith-linked service to others.
26	My religious leaders' opinions on vaccination matter to me.	SI	Social influences, family, peers, providers, and religious leaders	Religious leader influence	Retained to capture faith leader guidance as a trusted social influence.

No.	Final Q-set statement	Primary code	Primary domain	Attitudinal position	Selection rationale
27	I believe vaccine mandates infringe on personal freedom.	VM	Support for or opposition to vaccine mandates	Mandate opposition and autonomy	Retained to capture freedom-based objection to vaccine mandates.
28	I trust government health agencies to provide accurate information about vaccines.	TPH	Trust or distrust in public health agencies and scientific institutions	Trust in government health agencies	Retained to capture institutional trust in government health information.
29	I am more likely to trust vaccination information from sources that share my political views.	PI	Political and ideological influences	Politically aligned information trust	Retained to capture identity-aligned political information sources.
30	I am confident that vaccines are thoroughly tested before being approved.	SC	Safety concerns, testing, and side effects	Vaccine safety and confidence	Retained to capture confidence in vaccine testing before approval.
31	Scientists do not have the public's best interests in mind regarding vaccines.	TPH	Trust or distrust in public health agencies and scientific institutions	Distrust in scientists	Retained to capture suspicion toward scientists' motives.
32	I trust information about vaccines from official health organizations, such as the Centers for Disease Control and Prevention (CDC) or the World Health Organization (WHO).	TPH	Trust or distrust in public health agencies and scientific institutions	Trust in official health organizations	Retained to capture trust in organizations such as the CDC and WHO.
33	I believe that vaccine side effects are fully disclosed to the public.	SC	Safety concerns, testing, and side effects	Transparency about side effects	Retained to capture perceived disclosure of vaccine side effects.
34	I believe vaccines are promoted for profit, not for health.	SK	Skepticism about pharmaceutical or scientific motives	Pharmaceutical profit skepticism	Retained to capture skepticism about profit motives behind vaccine promotion.
35	I believe natural immunity is better than vaccine-acquired immunity.	VE	Vaccine effectiveness and public health value	Natural immunity preference	Retained to capture doubts about vaccine-acquired immunity compared with natural immunity.
36	I find it hard to know which vaccine information sources to trust.	VHA	Vaccine hesitancy, acceptance, and uncertainty	Source uncertainty	Retained to capture difficulty identifying trustworthy vaccine information.
37	The best way to teach vaccine-related issues is by showing the data.	VC	Vaccine communication, education, and interventions	Data-only teaching orientation	Retained to capture a simpler data-centered education position for comparison with item 1.

Table S5. Selection rules used during concourse reduction

Selection rule	How it was applied	Purpose
1. Domain coverage	Statements were grouped by content domain to ensure the final set covered trust, safety, religion, politics, social influence, mandates, misinformation, autonomy, public health value, and communication.	Prevented the Q-set from being dominated by only hesitancy or safety concerns.

Selection rule	How it was applied	Purpose
2. Spectrum balance	Within domains, statements were reviewed for acceptance, confidence, urgency, ambivalence, uncertainty, skepticism, autonomy-based caution, and refusal.	Responded to observed bias in the literature toward hesitancy and refusal.
3. Self-referential wording	Statements were preferred when participants could sort them from a first-person position.	Reduced literature-summary statements that were less suitable for Q sorting.
4. Clarity and single meaning	Statements with unclear wording, technical language, or double-barreled phrasing were revised or removed.	Supported more reliable participant interpretation during sorting.
5. Redundancy reduction	Similar statements were merged or represented by the clearest version.	Reduced burden while preserving conceptual coverage.
6. Pilot and expert review	Expert review and student pilot feedback were used to identify gaps, ambiguity, and imbalance.	Improved clarity, coverage, and balance before the final Q-sort.

Others

Kade- Version 1.3.1

Qsortware- www.qsortware.net.
